# Disrupted Redox Regulation and Inflammatory Response in Pyoderma Gangrenosum

**DOI:** 10.3390/life15040611

**Published:** 2025-04-06

**Authors:** Simona Roxana Georgescu, Clara Matei, Corina Daniela Ene, Cristina Capusa, Mircea Tampa, Madalina Irina Mitran, Cristina Iulia Mitran, Gheorghe Nicolae, Ilinca Nicolae

**Affiliations:** 1 Department of Dermatology, ‘Carol Davila’ University of Medicine and Pharmacy, 020021 Bucharest, Romania; srg.dermatology@gmail.com (S.R.G.); mircea.tampa@umfcd.ro (M.T.); 2 Department of Dermatology, ‘Victor Babes’ Clinical Hospital for Infectious Diseases, 030303 Bucharest, Romania; 3 Departments of Nephrology, ‘Carol Davila’ University of Medicine and Pharmacy, 020021 Bucharest, Romania; cristina.capusa@umfcd.ro; 4 Department of Nephrology, ‘Carol Davila’ Nephrology Hospital, 010731 Bucharest, Romania; 5 Department of Microbiology, ‘Carol Davila’ University of Medicine and Pharmacy, 020021 Bucharest, Romania; irina.mitran@umfcd.ro (M.I.M.); cristina.mitran@umfcd.ro (C.I.M.); 6 Faculty of Psychology, Babeș-Bolyai University, 400347 Cluj-Napoca, Romania; nicolaengheorghe@gmail.com

**Keywords:** pyoderma gangrenosum, acute phase proteins, IL-17A, Beta2 microglobulin, glutathione

## Abstract

Introduction. The pathophysiology of Pyoderma Gangrenosum (PG) involves altered innate and adaptive immunity, mutagenic and epigenetic changes, the autoinflammatory state, and the overexpression of cytokines. This study investigated the potential contribution of inflammation, redox signaling, and the immune system in the pathogenesis of PG. Materials and Methods. This case–control study included 36 patients with PG and 30 controls. We have determined the serum concentrations of acute phase proteins (C-reactive protein—CRP, alpha1 glycoprotein acid—AGPA, Albumin), interleukin-17A -IL-17A, β2 microglobulin-β2MG, reduced glutathione-GSH, oxidized glutathione- GSSG, the GSH/GSSG ratio, and hematological parameters (white blood cells-WBC, neutrophil-lymphocyte ratio-NLR, erythrocyte sedimentation rate-ESR) in patients with PG compared with controls. Furthermore, we have evaluated the variations in these markers before and after treatment in PG patients. Results. The serum concentrations of acute phase proteins (CRP, AGPA, and Albumin) and the IL-17A, β2MG, GSH, GSSG, and GSH/GSSG ratio were significantly different between the PG group and controls. Hematological parameters (WBC, NLR, and ESR), acute phase proteins (CRP, AGPA, and albumin), and IL-17A showed an exaggerated and persistent inflammatory response in patients with PG. In patients with PG associated with systemic diseases, the dysregulation of the biochemical events was more severe. Conclusions. The acute phase proteins, β2MG-MHC class I complex, and the GSH-GSSG system are unbalanced in PG. Our results could improve the diagnosis and our understanding of the pathogenic basis of PG.

## 1. Introduction

Persistent chronic inflammation can induce harmful effects in the human body and can contribute to the development and progression of many diseases [1,2]. Under these conditions, the immune system stimulates the activity of several inflammatory cells, induces the production of various cytokines, and creates a favorable environment for the development of an inflammatory condition [3,4]. PG is a neutrophilic, inflammatory, and non-infectious dermatological disorder characterized by skin lesions (papules, pustules, vesicles, and nodules), which rapidly progress to ulcers and necrosis [5,6,7]. The global incidence of PG is estimated at 3–10 cases per million population per year; in the USA, 58 individuals with PG per 1 million population; in the United Kingdom, the incidence rate of PG is 0.63 per 100,000 person-years [8,9,10]. The etiology of PG encompasses a wide spectrum of causes; 49% develop spontaneously, 27% after minor trauma, and 17% after surgery. Several clinical variants of PG have been identified: classic ulcerative, bullous, pustular, vegetative, superficial granulomatous, peristomal, and extracutaneous PG [8,9,10]. The pathophysiology of PG involves altered innate and adaptive immunity, keratinocyte apoptosis, mutagenic and epigenetic changes, the autoinflammatory state, altered neutrophil and T cell responses, cytokine overexpression, and inflammasome activation [9,10,11]. To date, approximately 50% of the cases of PG are documented as idiopathic disease; the rest of the cases are associated with an underlying systemic disease (cancer, autoimmune diseases, and autoinflammation) or occur within certain autoinflammatory syndromes, such as PAPA (pyogenic arthritis, PG, and acne), caused by abnormalities of prolines/serines/threonine phosphatase-interacting protein 1 -PSTPIP1; PASH (PG, acne and suppurative hidradenitis) caused by multiple repeats of the CCTG microsatellite in the promoter region of the PTSPIP1 gene; PASS (PG, acne conglobata, suppurativa hidradenitis, seropositive spondyloarthropathies) characterized by alterations in IL-1B expression—the genetic mutations are still unclear [12]; PAPASH (pyogenic arthritis, PG, acne, and hidradenitis suppurativa) a syndrome associated with mutations in *PSTPIP1* and *MEFV* genes which promote the high production of IL-1B by the inflammasome [13]; and SAPHO (synovitis, acne, pustulosis, hyperostosis, and osteitis) [8,14,15,16,17], in which effector T cells seem to play a central role by releasing certain cytokines, such as IL-12, IL-17, or IL-23 [18].

Patients with PG do not have an overall increased risk of developing malignancies, and a preexisting cancer diagnosis does not predispose individuals to PG. Previously identified links between PG and cancer include hematologic malignancies and solid organ tumors [11,19,20]. Although current data do not support an epidemiological association between PG and malignancy, both conditions are characterized by abnormal immune surveillance, chronic inflammation, cellular stress, increased lymphocyte turnover, and neutrophil chemotaxis in response to pro-inflammatory factors [8,10,21]. The severity of PG culminates in a microenvironment characterized by abnormal neutrophil trafficking, increased concentrations of tumor necrosis factor-α (TNF-α) and interleukins (ILs-1α, IL-1β, IL-6, IL-8, IL-12, IL-15, IL-17, IL-23, IL-36, CXCL1, CXCL10, CCL20, CCL3, and CCL5) [22], aberrant Beta2-microglobulin (β2MG) variations [15,23], and the promotion of a Th1 response [15,24,25]. In PG lesions, increased levels of IL-1, CXCL1/8 IL-36, and IL-17 isoforms with NETosis were revealed. Furthermore, the analysis of the inflammatory infiltrate has demonstrated the presence of B cells, macrophages, and fibroblasts. The potential involvement of autoantibodies targeting neutrophil extracellular traps (NETs) components and citrullinated proteins, along with an increased number of circulating plasma cells and the activation of type 1 IFN pathways as well as Th1, Th2, and Th17 polarization, contribute to the pathogenesis of PG. [8].

Recent findings indicate that PG has a strong correlation with inflammatory processes and systemic diseases [5,11,26]. The number of Th17 cells, IL-17 and its receptor, are overexpressed in PG lesional skin [24,27]. IL-17 promotes the release of chemokines which attract neutrophils and stimulate the secretion of IL-6, GM-CSF, and IL-8. Furthermore, IL-1, TNF-alpha, and IL-6 induces the expression of the β2MG gene [9,28]. β2MG acts as a subunit of the major histocompatibility complex class I (MHC-I), ensuring the structural integrity of MHC-I and facilitating the presentation of intracellular antigens to natural killer (NK) cells and CD8+ T cells [29]. β2-MG has been documented as a modulator of inflammation and as a potential regulator of the immune system and systemic autoimmunity [15,30,31].

The association between the redox status and inflammation is complex, as they are dynamic processes that are constantly changing. Chronic inflammation is characterized by persistent immunological activation, the secretion of cytokines and chemokines, and an increased production of oxidants. When the redox system is dysregulated, cells develop defense systems responsible for transforming free radicals into stable molecules [32,33,34]. The present study was designed to analyze inflammation markers (white blood cells—WBC, neutrophil to lymphocyte ratio—NLR, erythrocyte sedimentation rate—ESR, C reactive protein—CRP, alpha1 glycoprotein acid—AGPA, and interleukin 17 A—IL-17A), oxidants and antioxidants (albumin, reduced glutathione—GSH, oxidized glutathione—GSSG, and GSH/GSSG ratio), and immunomodulators (beta 2 microglobulin—β2MG) in PG patients and to assess the role of cellular stress, immune responses, and inflammation in the pathogenesis of PG. In addition, we have evaluated the variations in those markers before and after treatment in PG patients. The primary objective of our study was to evaluate a potential relationship between the blood markers of inflammation or oxidative stress and treatment response.

## 2. Materials and Methods

A total number of 36 patients with PG were selected and compared with 30 controls. We have included 12 patients with idiopathic PG and 24 patients with PG associated with systemic diseases (10 inflammatory bowel disease, 5 hematological disorders, 6 arthritis, and 3 renal cancer).

The patients were treated according to disease characteristics with topical or systemic corticosteroids or a combination of both. For the topical treatment we used high topical potent corticosteroids (clobetasol or intralesional triamcinolone acetonid. Systemic treatment included 0.5 to 1 mg/kg of oral prednisone. The duration of the treatment depended on the severity of the disease and the response to therapy.

We enrolled adults over 18 years. We excluded patients with hepatic and/or renal dysfunction (with abnormal urinary concentrations of β2MG) and patients with ulcerated lesions who did not meet the criteria for the diagnosis of PG.

The control group included healthy subjects, with no history of radiotherapy, chemotherapy, or systemic therapy and with no history of hematological or inflammatory diseases, malignancies, hepatic, or renal dysfunction (undetectable urinary β2MG, eGFR (mL/min/1.73 m^2^) > 90). All study participants signed an informed consent. The approval of the institutional ethics committee of the Victor Babes Hospital, Bucharest was obtained (no. 2/18 September 2017).

### 2.1. Biological Samples

Blood samples were collected within the first 2 days after diagnosis, as part of the pre-treatment assessment and after 6 months for post-therapeutic evaluation. After 30–60 min, the blood collected on the anticoagulant was used to perform hematological tests. The blood collected without anticoagulant was centrifuged (3000× *g*, 10 min) and aliquoted for current investigations or preserved at −80 degrees to determine specific markers. Samples of freshly emitted urine were used to investigate renal function.

### 2.2. Laboratory Determinations

Blood count was performed with an automatic hematology analyzer with 22 parameters—HMX (Beckman Coulter).

For the determination of ESR an automatic analyzer ALIFAX (SIREAnalytical Systems SRL, Udine, Italy) was used.

The serum concentration of hsCRP was measured using the latex immunoturbidimetric method. This method is based on an antigen–antibody agglutination reaction, where hsCRP in the sample reacts with anti-CRP antibodies bound to latex particles. The absorbance change, read at a wavelength of 570 nm using the HumaStar300 analyzer (Wiesbaden, Germany), is directly proportional to the hsCRP concentration in the sample. The results were reported in mg/dL.

The concentrations of orosomucoid were measured using an immunonephelometric assay with the MyBiosource MBS901995 kit (San Diego, CA, USA). The analysis was performed using the HumanStar300 analyzer (Wiesbaden, Germany), with readings taken at a wavelength of 340 nm. Results were expressed in g/L.

Serum albumin (peripheral venous blood collected a jeun) and urinary albumin were determined by colorimetric method, using bromocresol green (HUMASTAR3000).

Serum β2MG (peripheral venous blood collected a jeun) and urinary β2MG were quantified using an immunoturbidimetric assay with a latex-bound rabbit anti-β2-MG polyclonal antibody. We used the Beta-2 microglobulin Protein kit, Human (HEK293, His), and HumaStar300 analyzer. The linear measurement range is 0.2–8.0 mg/L and the reference range is 0.8–2.2 mg/L.

For the measurement of total glutathione/oxidized glutathione we used the GSH/GSSG Colorimetric Assay kit E-BC-KO97-M, Elabscience, Houston, TX, USA (detection range 0.36–30 μmol/L; sensitivity 0.36 μmol/L). It measures total, reduced, and oxidized glutathione in biological samples, using an enzymatic method requiring the Ellman reagent (DTNB, 5,5′-dithiobis-(2-nitrobenzoic acid) and glutathione reductase (GR). DTNB reacts with reduced glutathione to form a yellow product. The rate of change in optical density is directly proportional to the concentration of glutathione in the sample. For the evaluation of the final product, a semiautomatic analyzer was employed with a wavelength of 412 nm (Tecan GmbH, Grodig, Austria). The unit of measurement was μmol/L.

Serum IL-17A concentration was measured using a sandwich ELISA method (Elabscience, EL H0105, Houston, TX, USA). The assay had a detection range of 31.25–2000 pg/mL and a sensitivity of 18.75 pg/mL. Optical density (OD) was measured spectrophotometrically, with the OD value being directly proportional to the IL-17A concentration in the sample. A semi-automatic analyzer operating at a wavelength of 450 nm (Tecan GmbH, Grodig, Austria) was used to process the results. The final measurements were expressed in μmol/L.

### 2.3. Statistical Analysis

The Wilcoxon test was used to compare two groups. The relationships between pairs of parameters were analyzed using Spearman’s correlation coefficient, as determined by the results of the Kolmogorov–Smirnov normality test. A *p*-value below 0.05 was regarded as statistically significant.

## 3. Results

Table 1 summarizes the baseline study participant characteristics and response to treatment, which has been considered as a major study outcome. Approximately 52% of patients experienced a single PG lesion, and 48% presented multiple lesions of PG. A total of 27 patients presented ulcerative PG, while 7 patients had a bullous form, 1 patient had vegetative PG, and one patient had pustular PG. Lower limb involvement was documented in 26 patients, 2 patients presented the lesions on their trunk, 1 patient on his neck, and 7 on their upper limbs. The patients were treated according to the disease characteristics with topical or systemic corticosteroids or a combination. After a patient follow-up period of 6 months, complete ulcer healing was recorded in 24 patients and 5 achieved partial remission (Table 1)

The comparative analysis of the laboratory parameters, currently used in clinical practice as indicators of inflammation, in all PG patients and controls is presented in Table 2. Hematologic parameters (WBC, NLR, and ESR) and acute phase proteins (CRP, AGPA, and albumin) showed a persistent inflammatory response in PG patients compared to controls. The presence of inflammation in PG patients was also supported by the IL-17A concentrations, which were significantly increased in PG patients versus controls. The immune system reacts promptly, by mobilizing inflammatory cells in the affected area and increasing the release of β2MG in the circulatory system and deregulating the GSH-GSSG system in PG patients (Table 2). On the contrary, a decrease in anti-inflammatory factor concentrations, such as albumin and glutathione, was found.

We have divided the patients with PG into two groups, patients with idiopathic PG and PG associated with systemic diseases, and compared the concentrations of IL-17A, β2MG, GSH, and GSSG between the two groups. The comparison of the variations in IL-17A, β2MG, GSH, GSSG and the GSH/GSSG ratio between idiopathic PG and PG associated with systemic diseases indicates that cellular stress and inflammatory and immune responses are significant contributors to the pathophysiology of PG (Table 3).

The relationship between pretherapeutic serum concentrations of β2MG, IL-17A, and GSH/GSSG and the clinicopathological characteristics of PG patients was evaluated. The inflammatory and redox markers, studied in PG, showed significant correlations with the clinical and biological characteristics of PG patients (Table 4), which reinforces the involvement of inflammation, immune imbalance, and redox in the pathogenesis of the disease. We identified a strong negative correlation between IL-17A and GHS/GSSG and a strong positive correlation between IL-17A and AGPA as well as serum β2MG. Regarding serum β2MG, the correlations were similar. There was a negative correlation between the disease duration and GSH/GSSG. In addition, there was a positive correlation between the number of lesions and the serum β2MG concentration.

**Figure 1:**
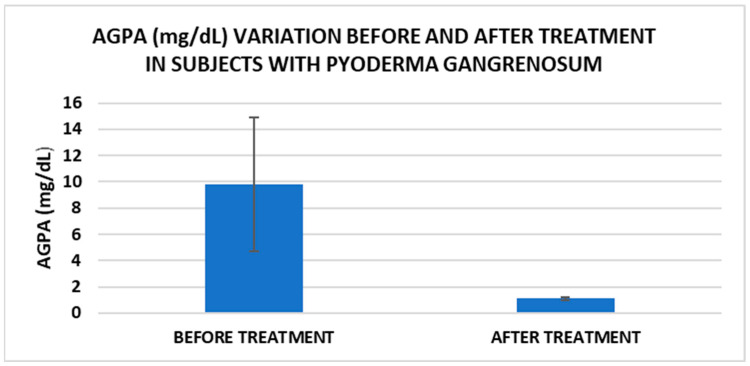
Treatment influence on AGPA concentrations (*p* < 0.001).

**Figure 2:**
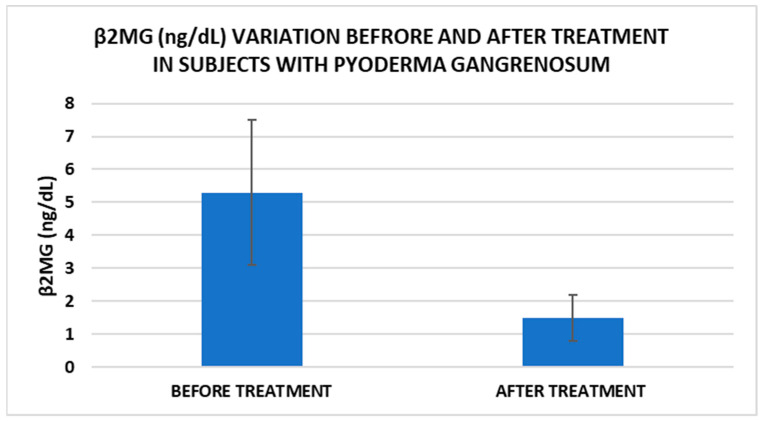
Treatment influence on serum β2MG concentrations (*p* < 0.001).

**Figure 3:**
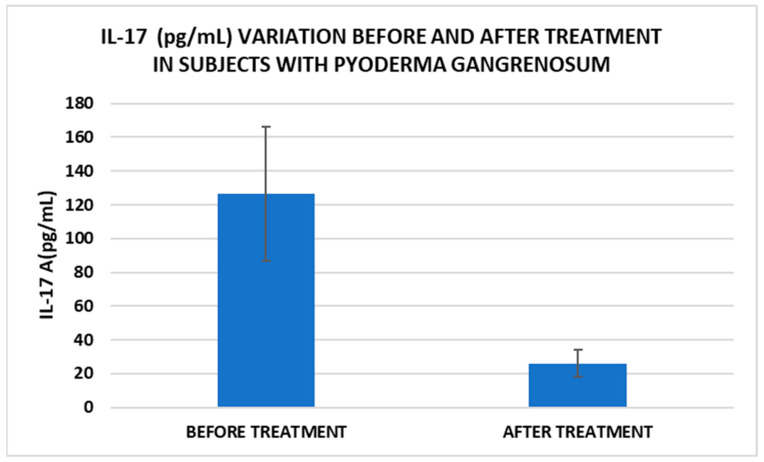
Treatment influence on IL-17 concentrations (*p* < 0.001).

**Figure 4:**
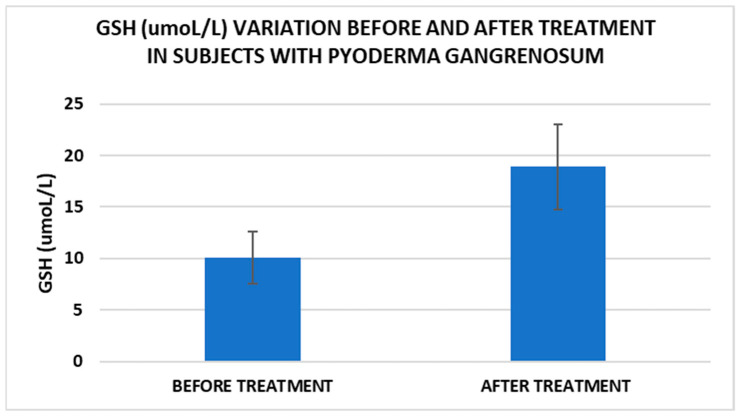
Treatment influence on GSH concentrations (*p* < 0.001).

**Figure 5:**
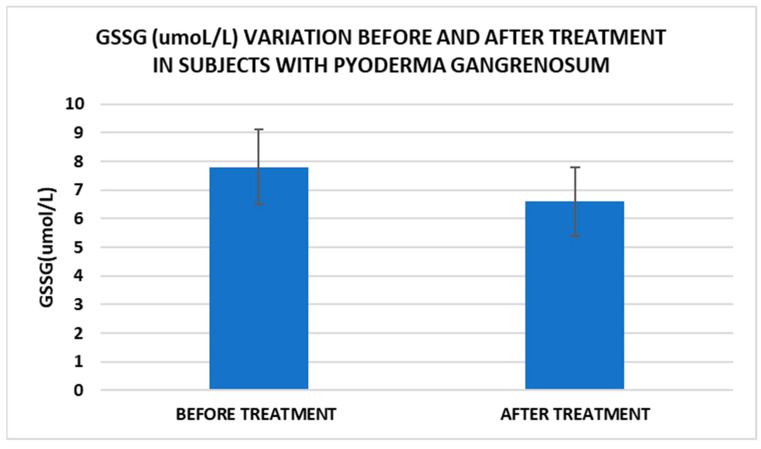
Treatment influence on GSSG concentrations (*p* < 0.001).

**Figure 6:**
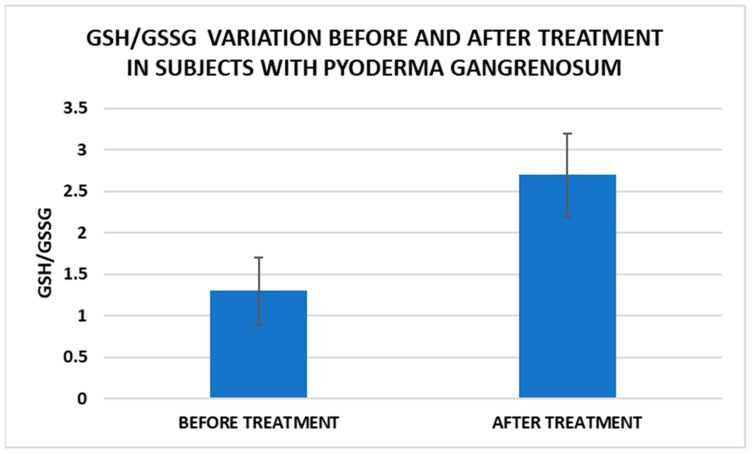
Treatment influence on GSH/GSSG concentrations (*p* < 0.001).

After treatment we observed the normalization of the concentrations of hematological parameters, acute phase proteins, and IL1-7A. It should be noted that the healing of PG lesions was accompanied by the restoration of the GSH cycle and the stability of the β2MG-MHC complex (Figure 1, Figure 2, Figure 3, Figure 4, Figure 5 and Figure 6).

## 4. Discussion

PG occurs as an isolated disease or in association with certain underlying systemic conditions [11]. This study investigated the potential contribution of inflammation, redox signaling, and the immune system in the pathogenesis of PG. The major dysregulated molecular pathways in PG, reported in this study, were as follows: the increase in oxidative stress and inflammation and the alteration of the β2MG production and redox signaling. The overexpression of hematological factors (WBC, NLR, and ESR), acute phase proteins (CRP and AGPA), and cytokines (IL-17A) associated with the decrease in anti-inflammatory factors (albumin and glutathione) could promote an exaggerated inflammatory response in PG patients (Table 2), which would explain the onset and rapid spread of PG lesions (Table 1). The inflammatory cascade in PG is based on an altered response of neutrophils, T cells, and inflammasomes; keratinocyte apoptosis; epigenetic changes [10,35]; and the up-regulation of the gene expression of acute phase proteins under the influence of cytokines [9]. The results obtained allow us to assume that the alteration of these biochemical pathways might be responsible for the slow wound repair in PG cases (Table 4). In conclusion, the current concept of acute phase reaction indicated a systemic response due to liver synthesis, complemented by a local response, modulated by cellular stress [1]. The alteration of the inflammatory response has been associated with the mitogen-activated protein kinase dysregulation (MAP3K7 and MAP3K5), caspase recruitment domain containing protein 9 (CARD9), upregulation of a signal transducer and transcription activator (*STAT1* and *STAT4)* genes, downregulation of the GATA binding protein 3 (*GATA3*) gene, and promotion of a Th17 response [10,15,24].

The association between inflammatory and circulating immune markers in PG has not been consistently analyzed. In line with the hypothesis that β2MG could play a role in immune surveillance and inflammation, in the present study we found significantly increased serum β2MG concentrations in PG patients versus controls (Table 2). There was a good positive correlation between β2MG and IL—17, as well as AGPA. There were medium positive correlations between β2MG, NLR, CPR, and the number of lesions (Table 4). Increased serum β2MG concentrations under autoinflammatory conditions can be explained by the upregulation of lymphocytes and mononuclear cells, which synthesize large amounts of β2MG. This process is regulated by interferon and pro-inflammatory monocytes and is a hallmark of PG [35]. Furthermore, a major expression of β2MG in PG patients facilitates the activation of NK and CD8 + T cells, facilitating the presentation of intracellular antigens. The complex β2MG/MHC class I exerts its biological roles by activating multiple intracellular signaling pathways (cAMP-dependent PAK activity; JAK/STAT, PI3K/AKT, PI3K/JNK, PI3K/ AKT/MYC) and interacting with some growth factor receptors ( IGFR, EGFR, IL-2R. IL-4R) and glucagon receptors [29,36]. In conclusion, β2MG, secreted by nucleated cells, modulates the immune and tumor microenvironment [10,29].

Cell metabolism is strongly interconnected with the redox state. When cellular homeostasis is affected by various factors, the redox system is dysregulated, leading to an imbalance between oxidation and reduction. To control the cellular stress, cells develop enzymatic and nonenzymatic defense systems, responsible for the transformation of free radicals into stable entities. The reductive system is inducible and finely regulated by two protection mechanisms, Nrf2-Keap1 and thiol/disulfide [4,37]. The endogenous enzymatic antioxidant system includes superoxide dismutase (SOD), glutathione peroxidase (GPX), glutathione S-transferase (GST), catalase (CAT), heme oxygenase (HO), peroxiredoxin (Prx, PRDX), thioredoxin (Trx), and paraoxonase (PON). The nonenzymatic antioxidant system includes GSH, NADPH, NADH, vitamin C, vitamin E, and melatonin. An increase in the ratio of GSH/GSSG, NADH/NAD+, NADPH/NADP+, or oxidized cysteine/cysteine (disulfide) leads to the restoration of the balance between oxidative stress and reductive stress [3,4]. In our study, the level of reduced GSH decreased and oxidized GSH concentrations increased due to oxidative stress generation in PG patients (Table 2 and Table 3). There were negative correlations between the GSH/GSSG ratio and inflammation intensity (evaluated through IL-17, CRP, AGPA, and ESR) and between the GSH/GSSG ratio and PG duration (Table 4). As a result, the GSH/GSSG ratio was used as an index of oxidative stress in the present study. Glutathione is a ubiquitous non-protein thiol involved in antioxidant defense, detoxification, cell proliferation, differentiation, and apoptosis [38]. In addition to glutathione, a large amount of sulfhydryl groups is found in proteins. In the current study, albumin concentrations were low in the pathologies we have studied (Table 2) and behaved as a weak antioxidant (Table 4). Albumin is essential in combating oxidative injuries. Redox signaling controls the function of the cell [38]. The therapy used in PG is aimed at reducing abnormal inflammation, the proper healing of skin ulcers, and the control of the underlying disease. In our study, the resolution of PG lesions was accompanied by the reduction in inflammatory mediators, improvement of the pro/antioxidant balance, restoration of the GSH cycle, and stability of the β2MG-MHC complex Figure 1, Figure 2, Figure 3, Figure 4, Figure 5 and Figure 6).

The comparison of variations in IL17A, β2MG, GSH, GSSG, and the GSH/GSSG ratio between idiopathic PG and PG associated with systemic diseases suggests that the second group experiences greater cellular stress, as well as more intense inflammatory and immune responses. There is also a progressive increase in the values of the markers studied, starting from the control group to the group with idiopathic PG and finally the group with PG associated with systemic diseases. The variations may also arise from the systemic disease itself, in addition to being part of the PG pathomechanism.

In our study, β2MG levels decreased significantly after treatment, which could be a marker to monitor the evolution of PG. Thus, for example, Tahir et al. reported a clinical case of a child diagnosed with PG who had very high levels of β2MG, which decreased dramatically after therapy. The authors suggest that this marker can be used for monitoring the disease but may also be useful for diagnosis [15]. Another marker with implications for PG management is IL-17. In our study, IL-17 levels decreased significantly after treatment. A very recent narrative review explored the available literature on cases where PG has been effectively treated with IL-17 inhibitors, as well as instances where PG may have emerged following their use. Based on the application of the Naranjo Adverse Drug Reaction Probability Scale, determining whether PG represents an adverse reaction to anti-IL-17 biological agents remains challenging [39].

In the current paper, we analyzed the relationship between the redox state, inflammation, and immunity in PG. These results support the hypothesis that the upregulation of oxidants is a cause of altered immune and inflammatory responses that lead to the development of PG [36,40]. Although the small sample size is a limitation of this study, promising results have been obtained on the disruption of some essential biological pathways, as a result of the use of suitable biological samples and representative groups that can represent the basis for larger studies. The vast majority of patients with PG included in the study had ulcerative PG, the number of patients with another type, pustular or vegetative, was very small, which did not allow an analysis according to the clinical type. The reactive species of oxygen, nitrogen, and sulfur affect the homeostasis and maintenance and modulation of the redox potential of a wide range of biological responses and events. Redox changes are communicated to cells by “hormesis mechanisms” [41]. Our results could improve diagnosis and provide a deeper understanding of the pathogenic basis of PG. Our results support the multifaceted role of the redox state and inflammation in PG.

## 5. Conclusions

**Figure 7:**
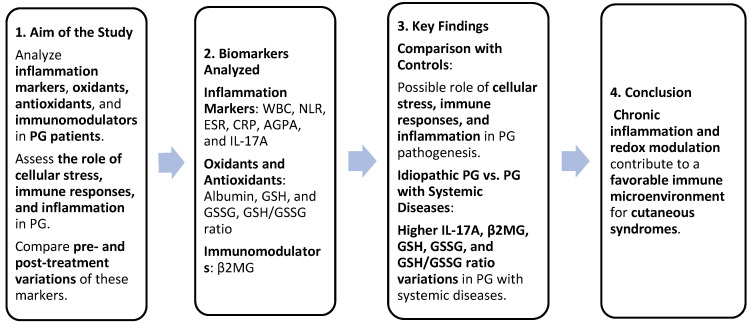
Summary of study design and findings. Greater cellular stress and stronger inflammatory/immune responses were identified in patients with PG associated with systemic diseases compared to patients with idiopathic PG. These findings could improve PG diagnosis and pathogenic understanding.

Serum variations in inflammation markers (WBC, NLR, ESR, CRP, AGPA, and IL-17A), oxidants, antioxidants (albumin, GSH, GSSG, and GSH/GSSG ratio), and immunomodulators (β2MG) between PG patients and controls confirmed the role of cellular stress, immune responses, and inflammation in the pathogenesis of PG. The comparison of variations in IL-17A, β2MG, GSH, GSSG, and the GSH/GSSG ratio between idiopathic PG and PG associated with systemic diseases suggested that the second group experienced greater cellular stress, as well as more intense inflammatory and immune responses. Chronic inflammation and redox modulation can create a favorable immune microenvironment for the development of cutaneous syndromes. Our results could improve the diagnosis of PG and our understanding regarding the pathogenic basis of PG (Figure 7).

## Figures and Tables

**Table 1:** The characteristics of the study participants.

Characteristic	Patients with PG (*n* = 36)	Controls (*n* = 30)
**Age (years)** Male/female ratio eGFR (mL/min/1.73 m^2^) Urinary β2 MG (mg/L) Albuminuria (mg/L)	56.7 ± 5.3 14/22 102.5 ± 12.3 0.10 ± 0.10 0.2 ± 0.3	50.7 ± 5.4 11/19 109. 5 ± 10.8 0.09 ± 0.11 s 0.1 ± 0.3
**Medical history** Idiopathic/associated with systemic diseases Size (cm) Number of lesions Disease duration (months)	12/24 6.3 ± 4.4 4.3 ± 3.4 3.5 ± 2.9	
**Subtype** Ulcerative Bullous Vegetative Pustular	27 (75.0%) 7 (19.4%) 1 (2.8%) 1 (2.8%)	
**Localisation** Lower extremities Head and neck Trunk Upper extremities **Clinical course** Explosive onset and rapid spread Indolent onset and gradual spread **Treatment options** Topical treatment Systemic treatment **Response to therapy (after 6 months)** Healed PG Partial remission of PG	26 (72.2%) 1 (2.8%) 2 (5.6%) 7 (19.4%) 33 (91.6%) 3 (8.3%) 20 (55.56%) 28 (77.78) 24 (66.66%) 12 (33.33%)	

PG, Pyoderma Gangrenosum; eGFR, estimated Glomerular Filtration Rate; and u β2MG-urinary Beta2-microglobulin.

**Table 2:** The laboratory data in PG patients and controls.

Parameter	PG Group (36 Cases)	Control Group (30 Cases)	*p* Value
WBC (×10^3^/mcL)	12.3 ± 4.4	4.4 ± 0.4	<0.001
NLR	2.98 ± 1.22	1.60 ± 1.01	<0.001
ESR (mm/h)	42.4 ± 20.2	4.5 ± 0.4	<0.001
CRP (mg/L)	78.7 ± 35.5	3.7 ± 3.5	<0.001
AGPA (g/L)	9.8 ± 5.13	0.80 ± 0.11	<0.001
Albumin (g/dL)	3.1 ± 0.9	4.2 ± 0.4	<0.01
serum β2MG (ng/dL)	5.3 ± 2.2	1.3 ± 0.4	<0.001
IL-17A (pg/mL)	126.6 ± 39.7	26.8 ± 7.3	<0.001
GSH (μmol/L)	10.1 ± 2.5	18.1 ± 0.9	<0.001
GSSG (μmol/L)	7.8 ±1.1	6.5 ± 0.9	0.034
GSH/GSSG	1.3 ± 0.4	2.8 ± 0.3	0.001

PG-Pyoderma Gangrenosum; WBC-white blood cells; NLR-neutrophil to lymphocyte ratio; ESR erythrocyte sedimentation spleen; AGPA-Alpha1 glycoprotein acid; CRP-C-reactive protein; GSH-reduced glutathione; GSSG-oxidized glutathione (Glutathione Disulfide); β2MG Beta2-microglobulin; and IL-interleukin.

**Table 3:** The laboratory data in PG patients.

Parameter	Idiopathic PG (*n* = 12)	PG Associated with Systemic Diseases (*n* = 24)	Control (30 Cases)	*p* Value
IL-17A (pg/mL)	114.1 ± 28.5	138.2 ± 27.0	26.8 ± 7.3	p1 < 0.01 p2 < 0.01 p3 < 0.01
serum β2MG (ng/mL)	4.9 ± 1.9	5.7 ± 2.0	1.3 ± 0.4	p1 < 0.01 p2 < 0.01 p3 < 0.01
GSH (μmol/L)	10.9 ± 2.2	9.2 ± 1.8	18.1 ± 0.9	p1 < 0.01 p2 < 0.01 p3 < 0.01
GSSG (μmol/L)	7.2 ± 1.7	8.4 ± 1.3	6.5 ± 0.9	p1 < 0.05 p2 < 0.01 p3 < 0.01
GSH/GSSG	1.5 ± 0.4	1.1 ± 0.3	2.8 ± 0.3	p1 < 0.01 p2 < 0.01 p3 < 0.01

p1—idiopathic—PG group vs. PG associated with systemic diseases; p2—idiopathic PG group vs. control group, p3—PG associated with systemic diseases vs. control group. PG—Pyoderma Gangrenosum; β2MG—Beta2-Microglobulin; IL—interleukin; GSH—reduced glutathione; and GSSG—oxidized glutathione (Glutathione Disulfide).

**Table 4:** Correlations between β2MG, IL-17A, and GSH/GSSG, and the clinical and biological characteristics of PG patients.

Parameter	Variable	Serum β2MG	IL-17A	GSH/GSSG
IL−17 (pg/mL)	Rho	0.87	/	/
	*p*	<0.01	/	/
GSH/GSSG	Rho	−0.68	−0.79	/
	*p*	<0.01	<0.01	/
WBC (×10^3^/mcL)	Rho	0.13	0.37	−0.26
	*p*	0.02	0.07	0.09
NLR	Rho	0.61	0.25	−0.11
	*p*	<0.01	<0.01	0.02
ESR (mm/h)	Rho	0.18	0.12	−0.37
	*p*	0.05	>0.05	0.05
CRP (mg/L)	Rho	0.52	0.19	=0.81
	*p*	<0.01	<0.01	<0.01
AGPA (g/L)	Rho	0.89	0.92	−0.58
	*p*	<0.01	<0.01	<0.01
Albumin (g/dL)	Rho	−0.37	−0.41	0.92
	*p*	0.03	0.05	<0.01
Disease duration (month)	Rho	0.12	0.18	−0.44
	*p*	>0.05	>0.05	<0.01
Number of lesions	Rho	0.64	0.11	0.12
	*p*	<0.01	0.02	>0.05
Size (cm)	Rho	0.09	0.26	0.13
	*p*	>0.05	>0.05	>0.05

PG—Pyoderma Gangrenosum; β2MG—Beta2microglobulin; IL—interleukin; GSH—reduced glutathione; GSSG—oxidized glutathione (Glutathione Disulfide); WBC—white blood cells; NLR—neutrophil to lymphocyte ratio; ESR—erythrocyte sedimentation spleen; AGPA—Alpha1 glycoprotein acid; CRP—C-reactive protein; *p*-Values Correlation in Linear Regression Analysis; and rho-Linear Regression Coefficient.

## Data Availability

All data are contained within the article.
